# Posterior cerebral artery embolism resulting in bilateral paramedian thalamic infarction: A case report

**DOI:** 10.1097/MD.0000000000032071

**Published:** 2022-11-25

**Authors:** Shaowei Xie, Ning Han, Xingyu Chen, Kuochang Yin, Guodong Xu, Yanhong Dong, Peiyuan Lv

**Affiliations:** a Department of Neurology, Hebei General Hospital, Shijiazhuang, Hebei, China; b Hebei Provincial Key Laboratory of Cerebral Networks and Cognitive Disorders, Hebei General Hospital, Shijiazhuang, Hebei, China.

**Keywords:** cerebral infarction, fluctuating consciousness, mechanical embolization, thalamus

## Abstract

Bilateral thalamic infarcts are not easily recognized, it have diverse clinical manifestations and relatively severe symptoms. It may leave long-term drowsiness, cognitive impairment, and speech impairment. We report a case of bilateral paramedian thalamic infarction with impaired consciousness as the main symptom. The digital subtraction angiography suggested that the left superior cerebellar artery and posterior cerebral artery (PCA) were occluded.

**Patients concern:**

A previously 67-year-old man was taken to our hospital after 9.5 hours of acute dizziness and loss of consciousness.

**Diagnosis:**

The cranial DWI + MRA suggested acute cerebral infarction in bilateral thalamus and bilateral midbrain, and the left posterior cerebral artery was not clearly visualized. The patient was diagnosed with posterior cerebral artery embolism.

**Interventions:**

A mechanical thrombectomy was performed.

**Outcome:**

The patient’s symptoms did not completely improve after revascularization, followed by fluctuating consciousness.

**Lessons:**

Recurrent lethargy in patients after endovascular treatment may be a clinical manifestation of damage to thalamic structures or due to the presence of ineffective recanalization.

## 1. Introduction

Bilateral thalamic infarcts are not easily recognized, it have diverse clinical manifestations and relatively severe symptoms. The most common clinical symptoms include bilateral vertical gaze palsy, memory impairment, and impaired consciousness.^[1]^ The paramedian thalamic blood supplied by the paramedian thalamic artery originated from the posterior cerebral artery (PCA). There is a rare anatomical type of paramedian thalamic artery in which the bilateral arteries originate from the P1 segment of the PCA on one side (type IIa variant). However, this type is rarely reported. Here, we report a case of bilateral paramedian thalamic infarction due to PCA occlusion. Interestingly, the patient’s symptoms did not completely improve after mechanical embolization and subsequently developed fluctuating consciousness.

## 2. Case presentation

A previously 67-year-old man was taken to our hospital after 9.5 hours of acute dizziness and loss of consciouseness. His past medical history was unremarkable, and no history of smoking or excessive alcohol consumption. Intravenous thrombolysis was performed before admission, but no effect. On admission, his physical examination were as follows: body temperature 35.5℃, heart rate 64 bpm, breathing frequency 16 bpm, blood pressure 150/80 mm Hg, neurological fundings: comatose state, Glasgow Coma Scale of 6 points (1 points for ocular, 1 points for verbal, and 4 points for motor components). Bilateral pupils diameter was 2.5 mm, but the left pupil irregular in shape and the light reflex was absent, the right pupil with a regular shape and a blunted light reflex. After painful stimulation, escape movements were visible in the limbs. Positive Babinski’s sign bilaterally. The National Institute of Health Stroke Scale score was 20 points.

**Figure 1.**
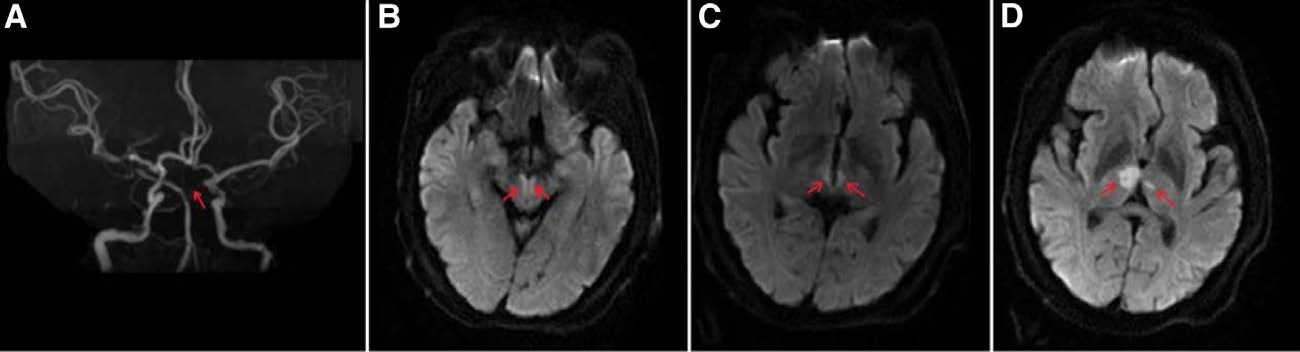
DWI + MRA imaging of the brain illustrates. (a) The left PCA is not clearly shown. (b) Hyperintense lesion of bilateral midbrain. (c and d) Hyperintense lesion of bilateral thalamic.

**Figure 2.**
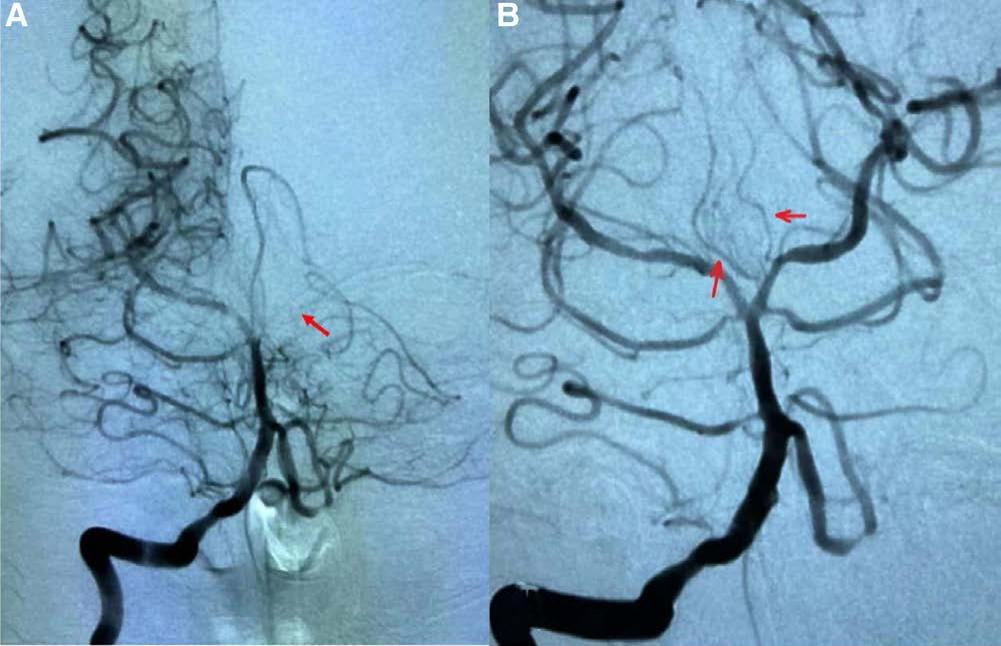
DSA imaging of intracranial arteries illustrates. (a) Occlusion of the left superior cerebellar artery and posterior cerebral artery. (b) After mechanical embolization, both bilateral paramedian thalamic arteries originated from the left posterior cerebral artery.

All blood tests were normal. Electrocardiogram and transthoracic echocardiography were unremarkable. The cranial DWI + MRA suggested acute cerebral infarction in bilateral thalamus and bilateral midbrain, and the left PCA was not clearly visualized (Fig. 1). The patient was diagnosed with PCA embolism, and immediately underwent mechanical thrombectomy. The digital subtraction angiography showed that the left superior cerebellar artery and PCA were occluded. After aspirate of the thrombus, the angiogram showed the bilateral superior cerebellar artery and PCA were complete revascularization (Fig. 2). The thrombolysis in cerebral infarction scale was grade 3 flow. The review of the cranial CT indicated no hemorrhage. Based on the imaging results, it was confirmed that bilateral thalamic infarction was caused by occlusion of the thalamic paramedian artery (type IIa variant).

On the third day after admission, the patient’s consciousness disorder improved, he can carry out simple conversation with others. But on the 4th day after admission, the patient became drowsiness. He can responded to painful stimulation, but could not open his eyes. And there was no significant change in the cranial CT scan. Later, the patient was intermittently drowsiness for a few hours to >10 hours, and could wake up by himself. The patient’s discharged to community rehabilitation hospital 12 days after admission. We followed up the patient 2 weeks after discharge, the duration of daytime sleep was significantly shortened, but he still had vertical gaze paralysis of both eyes and cognitive infaction.

## 3. Discussion

There have been many reports of bilateral thalamic infarction due to perchen artery occlusion (AOP). The imaging presentation and clinical symptoms of this case are very similar to those of AOP.

This patient had intermittent drowsiness as the main feature after mechanical thrombectomy, which was easily mistaken for infarct aggravation or hemorrhage, but there was no significant change on reexamination of CT compared with the previous one. This may be a normal clinical evolution after bilateral thalamic infarction. The sleep-wake cycle is regulated by complex interactions between different regions of the brainstem, hypothalamus, thalamus and preoptic area, and the thalamus plays an important role in sleep regulation and maintenance of wakefulness. Impairment of the arousal system underlies most post-stroke hypersomnias. Bilateral thalamic infarction interrupts the reticular formation of the midbrain and the superior reticular activation system projecting to the inner nucleus of the thalamus plate, so patients experience symptoms of hypersomnia. It has been shown that sleep-wake disturbances are more pronounced in bilateral thalamic infarcts than in unilateral thalamic infarcts and usually improve significantly within 1 year after stroke, with sleep requirements remaining higher than pre-stroke levels 1 year later. However, sleep architecture did not improve over time, and there was still an increase in stage 1 sleep and a decrease in stage 2 sleep and sleep spindle waves, which are the “sleep EEG features” of parthalamic median infarcts.^[2]^

The patient’s time from onset to diagnosis was 9 hours, which is an indication for endovascular intervention for acute ischemic stroke. However, the patient’s clinical symptoms did not completely improve after arterial recanalization. To analyze the reasons, the patient had a DWI-ASPECTS score of 6. Although it has been shown that the results of endovascular treatment in subgroups with DWI-ASPECTS scores of 4 to 6 compared with subgroups of 7 to 10 suggest no significant differences in terms of good prognosis, symptomatic intracranial hemorrhage, and mortality.^[3]^ And the good prognosis of patients with DWI-ASPECTS scores ≤ 6 in the successful reperfusion group was significantly higher than that of the medical treatment group.^[4]^ However, the ischemic semidark zone is the core of clinical treatment. For patients with ASPECTS score > 6 or onset more than 6 hours, in addition to improve CT and CTA, it is also recommended to improve cerebral perfusion imaging to clarify the perfusion-infarct core mismatch and weigh the advantages and disadvantages before endovascular treatment.^[5]^ While the patient in this case was not evaluated for perfusion-infarct core mismatch at the time of admission, which may be one of the reasons why the clinical symptoms did not improve completely after revascularization. Besides, the patient may has developed ischemia-reperfusion injury, and some studies have shown that baseline NIHSS score > 17, time from onset to revascularization >3 hours, and ASPECTS score <7 are predictors of ineffective revascularization.^[6–8]^

In summary, for patients is considered bilateral thalamic infarction, clinicians need to be more alert. They should pay attention to whether the occlusion of the starting segment of the PCA leads to a variant bilateral paramedian thalamic artery occlusion, which is important for finding the cause and subsequent treatment of patients. On the other hand, recurrent lethargy in patients after endovascular treatment may be a clinical manifestation of damage to thalamic structures or due to the presence of ineffective recanalization.

## Author contributions

Shaowei Xie and Ning Han collected the information, followed the patient, and wrote the paper; Xingyu Chen, Guodong Xu，Yanhong Dong，Peiyuan Lv revised the paper; Kuochang Yin was the patient’s doctor in charge; All authors read and approved the final manuscript.

**Conceptualization:** Shaowei Xie, Ning Han.

**Investigation:** Shaowei Xie, Ning Han.

**Methodology:** Shaowei Xie.

**Resources:** Kuocahng Yin.

**Writing – original draft:** Shaowei Xie.

**Writing – review & editing:** Xingyu Chen, Guodong Xu, Yanhong Dong, Peiyuan Lv.
